# Reducing the negative impact of accidents associated with the release of dangerous substances to environment

**DOI:** 10.3389/fpubh.2023.1270427

**Published:** 2023-11-03

**Authors:** Iveta Marková, Jozef Kubás, Zuzana Štofková, Katarína Petrlová

**Affiliations:** ^1^Department of Fire Engineering, Faculty of Security Engineering, University of Žilina, Žilina, Slovakia; ^2^Department of Crisis Management, Faculty of Security Engineering, University of Žilina, Žilina, Slovakia; ^3^Department of Economics, Faculty of Operation and Economics of Transport and Communications, University of Zilina, Zilina, Slovakia; ^4^Mathematical Institute in Opava, Silesian University in Opava, Opava, Czechia

**Keywords:** crisis management, risk management, quality of life, security, company, civil protection

## Abstract

**Background:**

The article is concerned with an evaluation of the current state of emergency readiness of industrial companies in the event of dangerous substance leakage and with a presentation of textile sorbents used for the purposes of capturing an escaped substance.

**Methods:**

A part of the article is concerned with the experimental designation of sorption capacity of hydrophobic, chemical, and universal sorption mats for chosen polar (water and alcohol) and non-polar (oil and gasoline) liquids. Experiments were realized according to Standard Test Method for Sorbent Performance of Adsorbents for use on Crude Oil and Related Spills, American Society for Testing and Materials (ASTM F726-17), type I. and Test methods for non-woven fabrics, European Union International Organization for Standardization (EN ISO 9073-6:2004). The aim of the article is an experimental designation of sorption capacity of textile sorption mats using two different methods, a comparison of the acquired results and a comparison of the acquired data with the data given by the manufacturer.

**Results:**

Textile sorbents, which can, owing to their sorption ability, allow the elimination or mitigation of a negative impact of a possible accident in the company connected with an escape of a liquid dangerous substance were tested and compared with the established values. Based on the obtained results it is possible to state that sorption capacities of the chemical and universal mat for the substrate water are equal and consistent with the data given by the manufacturer. Textile sorption mats also have a comparable sorption capacity. The sorption capacity on the substrate gasoline is the same in all textile sorbents. The adsorption capacity per unit mass all type’s sorbents was similar for non-polar liquids (gasoline was values from 6.41 to 6.57 and oil was values from 9.54 to 10.24).

**Conclusion:**

The acquired results confirmed the universality of textile sorption mats for gasoline. Sorption capacities of the chemical and universal mat for the substrate water are equal and match the data given by the manufacturer. Textile sorption mats have a maximum sorption output up to 60 s, afterwards the sorption capacity values remain unchanged.

## 1. Introduction

Crises are events which, in the most fundamental way, affect modern societies, endanger human lives, economies and social values. They are demarcated by wide-spread uncertainties and high stakes, as well as serious restrictions in the time available for analysis and reaction (1). Crises lead to severe consequences for societies and organizations (2). They usually occur due to various causes, such as natural catastrophes (earthquakes, wildfires, dust storms and extreme colds), technical and technological catastrophes (process fires, explosions, and leakages of toxic substances) or humanitarian actions (acts of terrorism, sabotages, violence, and strikes). Regardless of the cause of crises, each company or organization have to have a specific system of crisis management for minimizing property loss and loss of human life (3–5).

Nowadays in complex societies, management of wide-spread crises involves an enormous number of sectors, organizations, and individuals, which each have their own cultures, characteristics, assignments, and goals (1, 6, 7).

Industrial branches are complicated due to the number of elements/components, degree of uncertainty and a high degree of interaction between components (8). Taking into account that the creation of crises cannot be avoided, companies with operations involving a high level of risk have to find ways how to manage crises caused by their activity (9). Crisis management therefore has a large significance in these complex systems, because it helps mitigate the consequences of these large accidents and catastrophes (10).

Therefore, a crisis arises when the demands of the triggering event exceed the ability of a company, community, or organization to react, which leads to an endangerment of its continuation. Crisis management is then considered a “process of preparing for, reacting to and learning from the effects of a wide spectrum of large failings, which have an impact on groups of people, from organizations up to local, national and international communities” (11).

During crises, agility, and adaptability of these components of crisis management is decisive for the achievement of an efficient and effective reaction (12, 13) Incidents and catastrophes have broader, more long-term effects, which means that companies have to constantly adapt to challenges created by these effects and react to them (14).

A system of crisis management includes four phases: mitigation, readiness, reaction, renewal (15). The mitigation phase regards an effort to prevent future events/threats or to minimize their unfavorable effects. The readiness phase relates to an ability of a system to predict future events/threats and to offer the desired resources (16). The reaction phase is connected with an ability of a system to react to crises and to face unforeseen situations (17). Finally, the renewal phase regards proceedings necessary for a return to a normal state after the crisis occurs (16).

Based on sources about emergency readiness, crisis management consists of four correlated factors: prevention, preparation, reaction, revision (18). These factors are included in the commonly used three-stage approach, which describes crisis management as encompassing three phases. Before-crisis phase (prevention and preparation), crisis phase (reaction) and after-crisis phase (learning and repeating) (18).Currently, more and more studies are being carried out to assess the risks and determine their negative consequences on humans, with a quantitative assessment of the resulting consequeances (19, 20).

The final goal of a crisis management system is to be ready to solve disruptive incidents or critical situations in a rapid, appropriate and adequate manner (21). To minimize the effect of accidents and mitigate the unfavorable effects of emergency situations, systems of crisis management play an important role in managing emergency situations (22). In companies working with dangerous substances, preparing for crisis situations is essential. As several authors state, it is necessary to predict the scope of a possible negative event and to subsequently allocate adequate material to it, that will ensure absorption of a possible escaped substance (23). In the case of an escape of a liquid, sorbents are one of the possible solutions.

Sorbents are solids used in the separation of components from liquid mixtures (liquids and gases). They are classified according to chemical composition, structure (bulk, textile), origin (natural, synthetic), ability to sorb polar or non-polar compounds and chemicals. Natural adsorbents (sawdust, peat, sand, powdered sulphur, coal dust, etc.) have a lower absorption capacity compared to synthetic ones. Adsorption is considered to be the most widely used and safe environmental protection in the event of a spill of a hazardous substance. The concept of green adsorbents has been introduced to increase the safety of sorbent use, which means the recovery of cheap materials derived from, for example, agricultural waste (24, 25). Clay, whose sorption advantages have been investigated in several studies, can also be considered as a suitable and sustainable source of sorbents and has been shown to be effective in the sorption of radionuclides. Clays are widely regarded as suitable natural sorbents for wastewater decontamination and as a potential barrier in landfills to prevent and stop the leaching of radioactive material/radionuclides into bedrock and groundwater (26, 27). In the event of a spill into water, further response by emergency responders is required to prevent water toxicity, whereupon other special sorbents can be used, but the purpose may not be entirely effective (28–30).

Water pollution is a problem for human populations and also threatens ecosystems and habitats around the world. Contaminants in the environment may look unsightly and smell bad, but their negative impact goes beyond aesthetics. Some pollutants resist decomposition and accumulate in the food chain. These pollutants can be eaten or absorbed by fish and wildlife, and in turn can be consumed by humans. If water pollution causes algal blooms in a lake or marine environment, the proliferation of newly introduced nutrients stimulates plant and algal growth. This in turn reduces oxygen levels in the water and the lack of oxygen, known as eutrophication, smothers plants and animals which can create ‘dead zones’ where the waters are essentially devoid of life. In certain cases, these harmful algal blooms can produce neurotoxins that affect wildlife (31, 32). Therefore, an initial response with sorbets is needed to prevent the hazardous substance from coming into contact with the water or the environment.

Bulk sorbents are solids of different chemical compositions. They are designed to have the largest possible surface area, particularly suitable for removing thin layers of liquids over a large area. Their disadvantage is dustiness. Their use causes clogging (dusting) of the environment. On the other hand, they have long-term storage limits, being stored in bales. Also, the manipulation of these sorbents can cause occupational injuries during their application. For these reasons, such sorbents may not always be suitable for industrial plants and tend to be replaced by fabric sorbents. Therefore, their sorption properties should also be investigated, which contributes to a more effective response to potential spills of hazardous substances (33–36).

Textile sorbents absorb a substance on the basis of adhesion of the spilled liquid to the surface of the sorption material. They are demarcated by their ability to soak the escaped substance up in amounts that are many times larger than their own mass. They are made of polypropylene (PP) microfibers with a special property in regard to the kind of textile sorbent. Hydrophobic sorbents, as their name suggests, repel water. They are mainly used in leakages of substances on the surface of water. Thanks to their hydrophobia, they can float on the surface of water and mainly absorb escaped substances (in case of the escaped substance having a lower density than water, and therefore coating the surface of the water) (37). Universal sorption mats, similar to chemical mats, mainly bind water.

Universal sorption mats can be divided according to their ability to bind chemical substances into chemical, hydrophobic and universal ones. Each of these three groups has a typical color associated with it for an easy recognition of the sorbent (38).

**Figure 1:**
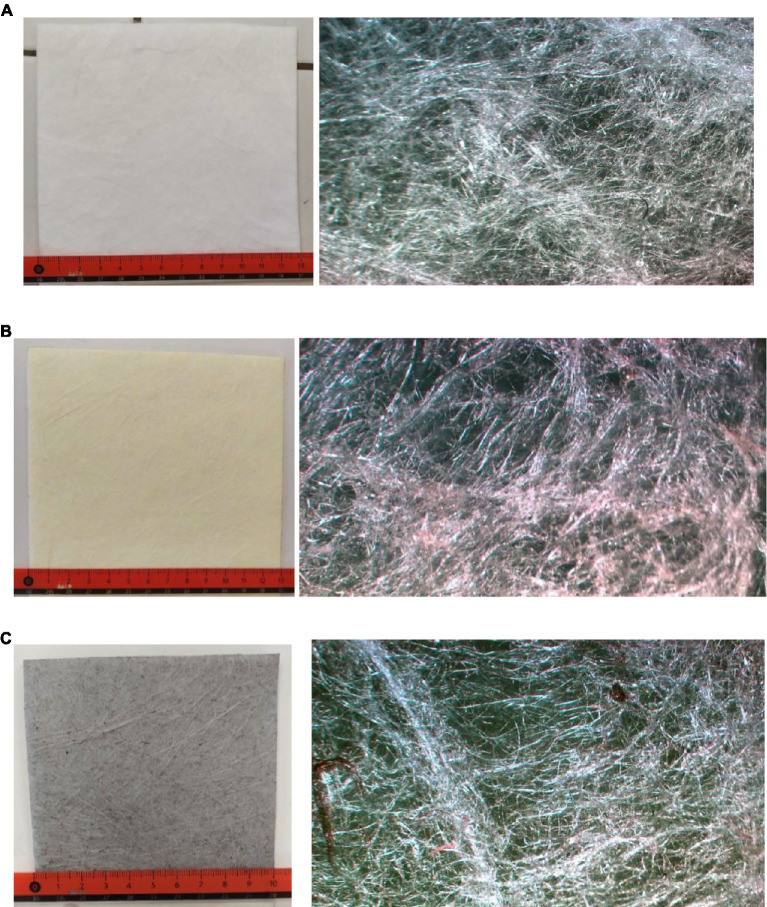
An example of experimental samples sorption mat before the experiment. **(A)** Hydrophobic sorption mat with microscope picture from Microscoph Olympus, 40x. **(B)** Chemical sorption mat with microscope picture from Microscoph Olympus, 40x. **(C)** Universal sorption mat with microscope picture from Microscoph Olympus, 40x.

Hydrophobic sorbents, as their name suggests, repel water. They are mainly used in leakages of substances on the surface of water. Thanks to their hydrophobiac nature of the sorbent, they can float on the surface of water and mainly absorb escaped substances (if the escaped substance has a lower density than water, and therefore coats the surface of the water). Hydrophobic sorption mats are predominantly of a white color (Figure 1A). Hydrophobic sorbents are made of polypropylene (PP) microfibers, thanks to which they are light-weight, even after a full soakage they do not fall to the bottom of the water container. Aside from lightness, microfibers also provide a high sorption capacity and escaped substances stay bound to the sorbent for a long time, do not spontaneously unbind, but should the need arise, the sorbate can be partially recovered (38). A hydrophobic sorbent can bind/absorb non-polar oil liquids. The hydrophobic sorbent does not bind water into its structure and therefore can float on the surface, it is usually used as a mat, floating barrage, and a sorption coil (39, 40).

Chemical sorbents are used mainly for the sorption of aggressive liquid chemical substances, such as hydroxides and concentrated acids. Chemical textile mats are often of a pink or yellow color (Figure 1B). Chemical textile sorbents are used in many different chemical laboratories or chemical plants, but they also appear in the equipment of emergency kits used by firefighting and emergency forces. The production of chemical textile mats is supplemented by a special hydrophilic modification of polypropylene (later just PP) microfibers. This modification influences their high resistance to aggressive chemical substances. Thanks to polypropylene they are highly resistant to abrasion, which allows for a simple manipulation after the sorption finishes. Similar to hydrophobic sorbents, they are demarcated by their high sorption capacity (Table 1) (39).

**Table 1:** Description of tested samples of textile sorption materials (41–43).

Textile sorbent	Label	Chemical composition	Description	Properties	Bio-degradability	Package weight (kg)	AC water	AC oil (l)
Hydrophobic sorption mat	HSR	100% polypropylene (PP)	White fibers	Non-toxic	No/hydrophobic	3	0	108
Chemical sorption mat	ChSR		Yellow fibers		No/hydrophilic	7.2	72	139
Universal sorption mat	USR		Gray fibers		No/hydrophilic	6	60	108

Universal sorbents, as their name already gives away, can be used universally, but they are not hydrophobic. They bind weak acids and water solutions, emulsions of fat, oil, and oil substances. A universal sorption of substances is facilitated by their composition. They are used in plants where various kinds of liquid substances are utilized. Universal sorption mats are usually of a gray color (Figure 1C).

Both chemical and universal sorbents are hydrophilic sorbents, materials that can bind water, hence both polar and non-polar liquids, to their hard surfaces. They are used in capturing dangerous substances – liquids- on a solid surface (44, 45).

The aim of the article is an assessment of the risk of dangerous substance leakage in an industrial accident on a company’s premises and a description of resources for effective capture of an escaped dangerous substance. After the acceptance of risks of dangerous substance escape follows a description and analysis of the application of textile sorption materials for a quick and effective capture of escaped substances, given polar and non-polar liquids, as a reaction to the arisen situation. At the same time, the goal is to establish the adsorption capacity of the given textile sorption materials by two different methodical approaches.

## 2. Materials and methods

### 2.1. Experimental samples textile sorbents

Adsorption is the ability to bind a gaseous or liquid substance with the surface layer of another solid substance. It is a physical event taking place at the phase interface liquid – solid phase or gas – solid phase, during which one or more components of the liquid or gas phase are concentrated on the surface of the solid phase of the adsorbent. The expression of the amount of adsorbed substance on the surface of the adsorbent is given by the parameter sorption capacity. The standard test method for sorbents for the adsorption test (ASTM F716-18) indicates the sorption capacity in %. The given data differs from the “dynamic sorption capacity,” which is given in weight units, so how many weight (mass) units of the given pollutant is the sorbent able to capture, or neutralize, if its sorption capacity is exhausted (give in g/g).

Adsorbents are solid substances used in the separation of components from liquid mixtures (liquids and hases). They are divided according to chemical composition, structure (loose, textile), origin (natural, synthetic), ability to sorb polar or non-polar compounds and chemicals. Natural adsorbents (wood sawdust, peat, sand, powdered sulfur, coal dust, etc.) have a lower absorption capacity compared to synthetic ones.

The ASTM F726 norm divides sorption materials into the following categories: type I., type II., type III. a, b; type IV. For this research, samples of type I. sorbents (46) were used, that is samples of sorbents whose length and width many times exceed their thickness, but simultaneously ones with linear character and a sufficient thickness, for example tapes and mats (Table 1).

The hydrophobic sorption mat (Figure 1A) is well suited for leakages of oil substances on both solid and liquid surfaces. The universal sorption mat (Figure 1A) is hydrophilic. Its sorption capacity for oil is the same as the sorption capacity of the hydrophobic mat. The chemical sorption mat (Figure 1B) states the highest sorption capacity values for both oil and water (41–43).

### 2.2. Adsorbed material – samples of polar and non-polar liquids

The research of sorption resources is realized according to established standards. The substrate on which the application of the sorbent is being realized and on which adsorption capacity is observed is oil. The discussed project is focused on evaluating the risk of an escape of dangerous substances in an industrial company as a result of an industrial accident. The chosen substances for the purpose of sorption are two polar solvents (water and alcohol) and two non-polar ones (gasoline and oil) (Table 2).

**Table 2:** Description of substances chosen as substrates for adsorption (47–49).

Substrate sample	Polarity of liquid	Density (g.cm^−3^)	Flash point (°C)	Explosion limit	Viscosity(mm^2^.s^−1^)
Oil	Non-polar	0.975 at 15°C	80	X	4 at 40°C
Car gasoline Super 95		0.750 at 15°C	−25	0.6–8 vol %	1 at 37.8°C
Water (data not specified)	Polar	1.000	Non-flammable liquid		0.896 at 25°
Ethanol		1.040	14°C – closed beaker	3.3–19	X

Oil is a part of testing standards (50, 51). Water and gasoline were chosen as polar liquids, being commonly used and gasoline also being the most commonly transported dangerous substance in our country (52). Gasoline and water are easily volatile liquids, but despite the stated fact, they were used for research purposes.

All experiments were realized during equal atmospheric conditions. All substrates were used for testing of sorption of the observed loose adsorption materials.

### 2.3. Experimental methods

**Figure 2:**
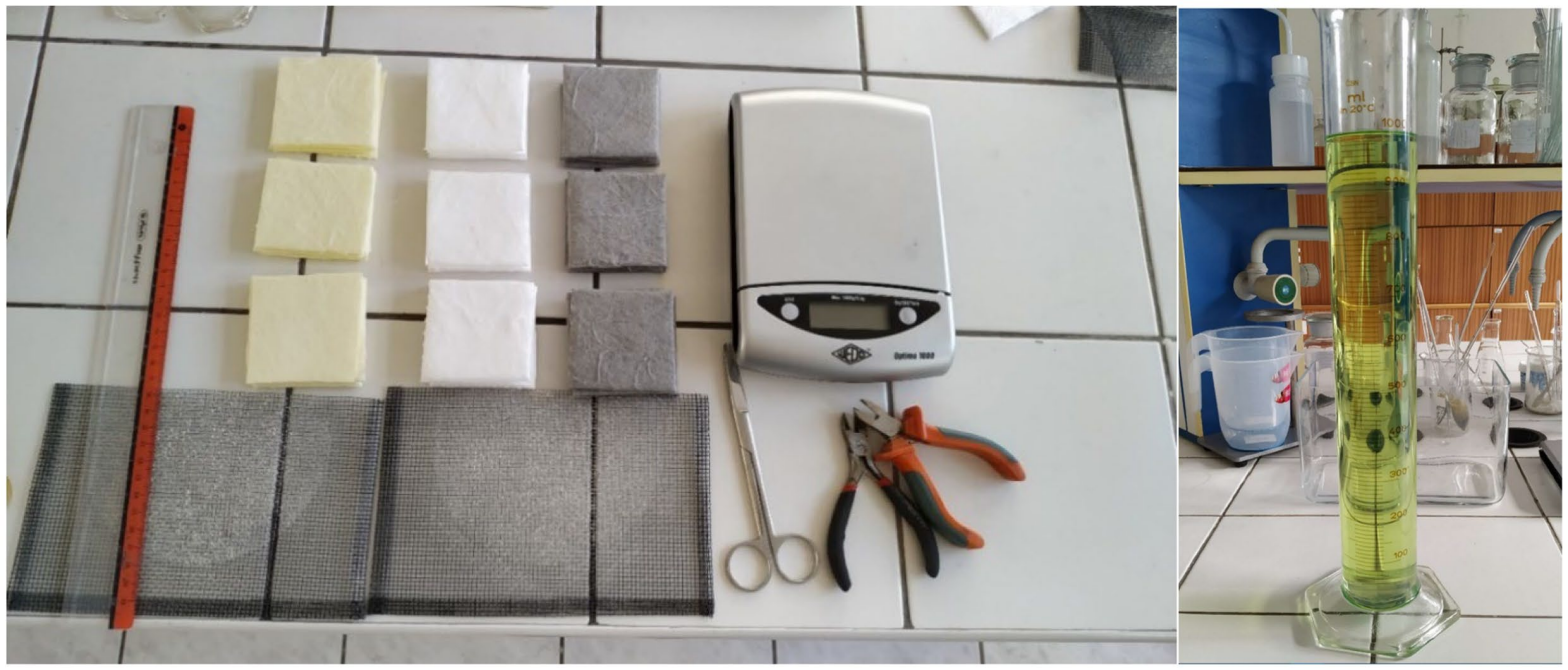
Tools for experimental determination of sorption capacity under atmospheric conditions.

Textile sorption resources can be tested by two standards for similar basic (Figure 2). In the Table 3, the conditions of the experiment are stated. The diversity of conditions was used to acquire relevant results.

**Table 3:** Experimental conditions of determining the sorption capacity of textile sorption mats (46, 53).

	ASTM F726–17 type I.	STN EN ISO 9073-6:2004
Size of sample	1300 × 1300 mm	1000 × 1000 mm
Mass minimum	4 g	1 g
Amount of liquid substance	Min. w. level = 2.5 cm	Min. w. level = 2 cm
Temperature	23 ± 4°C	20 ± 2°C
Air humidity	70 ± 20%	65 ± 4%
Soaking time	24 h	60 s
Sorption type	Adsorption	Absorption
Formula	$\left(a_{1}=\frac{m_{1}}{m_{4}}\;\right)$	$\mathrm{LAC}=\frac{m_{n}-m_{k}}{m_{k}}*100$

Time plays an important role in researching the effectiveness of sorbents. The sorption time for a liquid represents the time required for a sample of sorbent material to be completely wetted by a liquid medium, that is, for the liquid to penetrate its internal structure under the specified conditions (Table 3).

### 2.4. Determining the sorption capacity of textile sorbents according to ASTM F726

**Figure 3:**
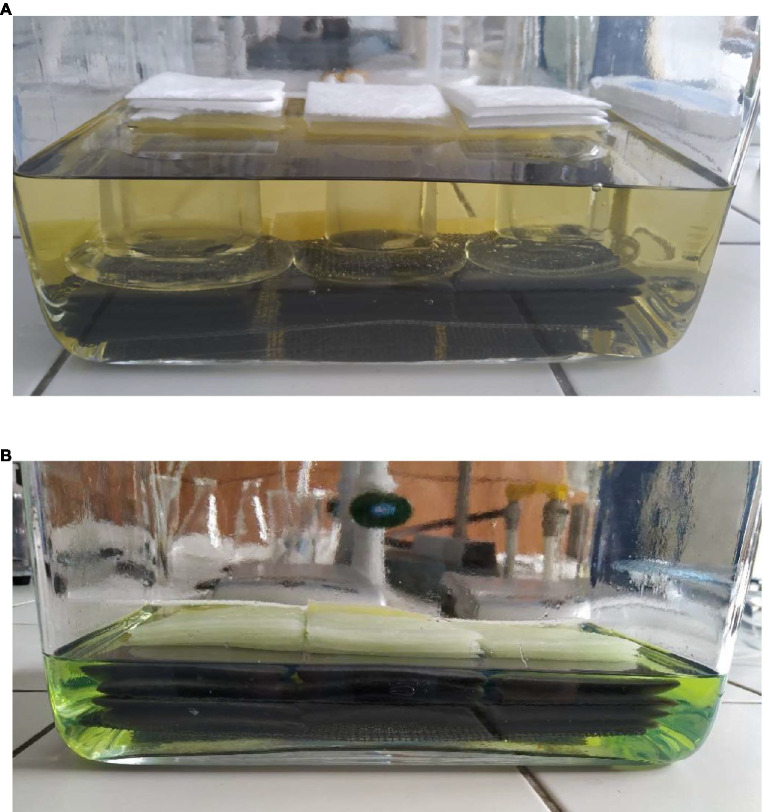
The soaking of textile mat samples in gasoline. **(A)** Beginning of sorption according to ASTM F276 at an ambient temperature of 23.2°C and air humidity 58%. **(B)** After of sorption according to ASTM F276 after 24 h of sorption, at an ambient temperature of 23.2°C and a humidity of 58%.

The standard sorbent testing method chosen is used in an adsorption trial in possible escapes of unrefined oil. Square-shaped samples (1,300 × 1,300 mm) (Figure 2) must have the required amount, which is determined based on the thickness and mass of the sample. The thickness of the sample must be 2.5 cm at minimum. If the sample is not thick enough, individual samples are layer on top of each other. Another limiting parameter is mass, which must be at least 4 g. The process is repeated three times. Prepared samples with the desired size, thickness and mass are inserted into a container filled with a given dangerous substance (Figure 3A). The sorption process is realized over 24 h ± 30 min (Figure 3B).

After the given time limit, the soaked sample is removed from the container and is affixed vertically, for the purposes of capturing the sorbate. After 30 ± 3 s (or 120 ± 3 s in a very viscous oil), the mass of the soaked sample is recorded. The adsorbed amount of the substance is calculated using the Eq. 1:


(1) $$m^{1}=m^{2}-m^{3}-m^{4}$$

where: m^1^ is the mass of the sorbent with sorbate [g], m^2^ the mass of the wet testing sample holder, crystallization dish and wet sorbent [g], m^3^ the mass of the wet sample holder and dish [g], m^4^ the mass of the dry sorbent [g].

The sorption capacity a1 [g] is calculated using the Eq. 2


(2) $$\mathrm{AC}=\frac{m^{1}}{m^{4}}$$

where: AC – maximum sorption capacity, m^1^ – mass of the sorbent with sorbate [g], m^4^ – mass of the dry sorbent [g].

### 2.5. Determining the sorption capacity of textile sorbents according to EN ISO 9073-6:2004

The EN ISO 9073-6 norm describes methods of evaluating select behavior properties of non-woven textiles when exposed to the effects of liquids, including adsorption capacity (A) of sorption material. All substances and sorption materials were conditioned at a temperature of 21°C and an air humidity of 65% for 24 h.

Textile mats (of a size 100 mm x 100 mm) and a minimum mass of 1 g were placed on a wire rack and inserted into a glass container with a chosen liquid (Figure 2). The samples were submerged approximately 20 mm below the surface of the chosen liquid for a duration of 60 s ± 1 s. The samples were subsequently left to vertically dry for a period of 120 s ± 3 s and their weight was measured. Experiment was repeated five times. The calculation of sorption capacity was realized according to the Eq. 3:


(3) $$\mathrm{LAC}=\frac{m_{n}-m_{k}}{m_{k}}*100$$

where: LAC is the adsorption capacity [%], m_k_ the mass of the dry testing sample [g], m_n_ the mass of the soaked sample at the end of the trial [g].

## 3. Results and discussion

The research of textile sorption materials is focused on observing:

- The time of sorption of a given liquid.
- The capacity of the sorption material.

The chosen experiment has stated the time of sorption in its methodology. The aim was to observe sorption capacity. Experimental results from individual methodical approaches are significantly comparable (Tables 4, 5).

### 3.1. Evaluating textile sorbents according to EN ISO 9073

**Figure 4:**
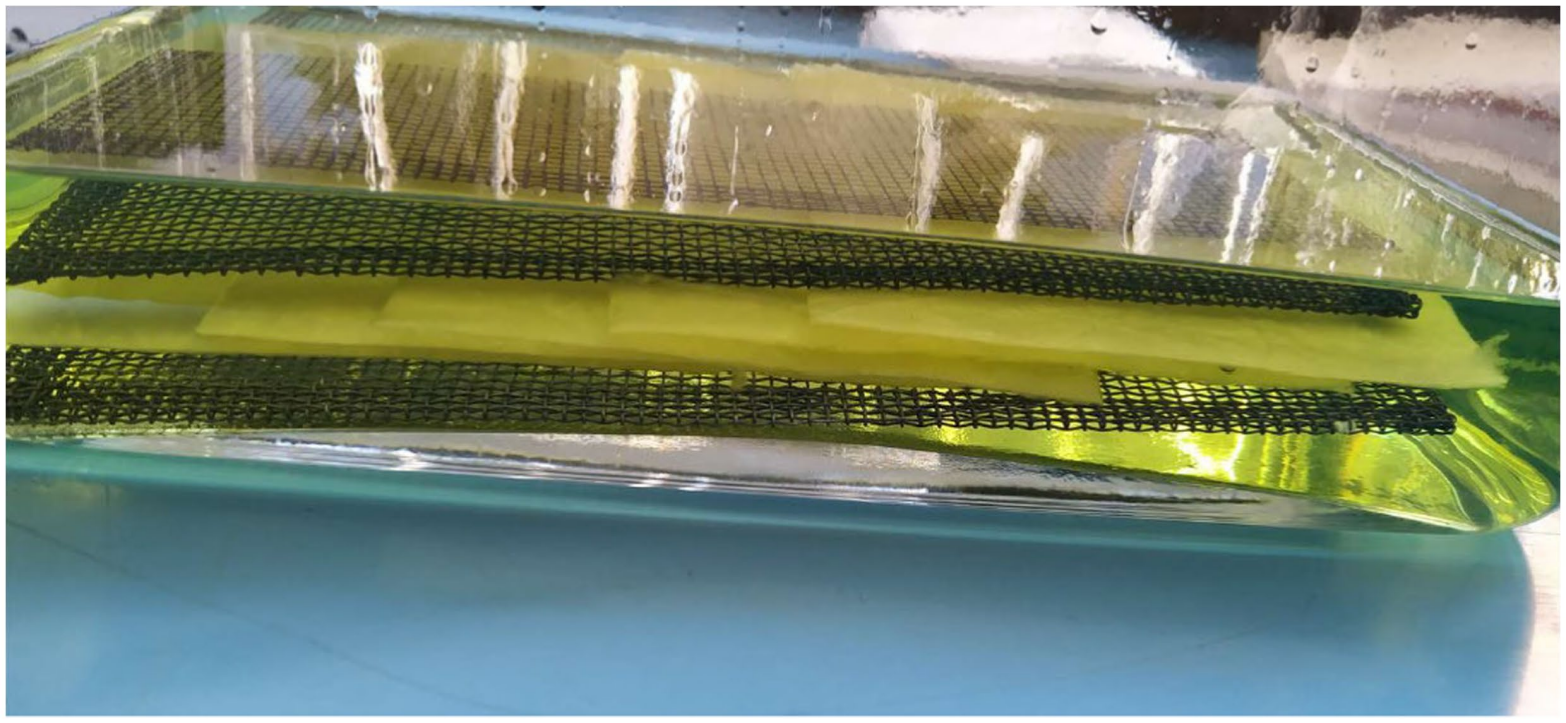
The soaking of textile mat samples in gasoline – beginning of sorption according to EN ISO 9073-6 after 60 s, ambient temperature 23.2°C and air humidity 58.

The sorption capacity established according to EN ISO 9073 has been observed over 60 s. The results were compared to the data given by the manufacturer (Table 6). The manufacturer states the sorption capacity for water and oil only (Figure 4). The manufacturer does not state the time of sorption. A match is proven in the sorption capacity for water (Figure 4A). The data acquired for oil are Figure 4B.

**Table 6:** Experimental results and a calculation of AC for water according to EN ISO 9073.

Textile sorption mats	Area of sample (cm^2^)	Average mass of dry sample (g)	Amount of absorbed water (g)	(*m_n_-m_k_*) given by the manufacturer (Technical sheets)	LAC	LAC given by the manufacturer (Technical sheets)
	Sorbate: water					
HSR	100	2.78 ± 0.0074	0.47 ± 0.098	0	0.17 ± 0.03	0
ChSR		1.88 ± 0.009	17.56 ± 1.14	10 mL/1 g	9.34 ± 0.95	10
USR		2.74 ± 0.33	27.28 ± 3.47	10 mL/1 g	9.93 ± 0.18	10

The results stated by the manufacturer seem deceiving because they are given in liters. The statement of sorption capacity must be explained as an amount (volume) of the received sorbate (liquid) per unit weight. The given approach does not present an adsorption capacity value, but rather the amount of absorbed volume of liquid denoted as (*m_n_* – *m_k_*). The stated parameter may be given in milliliters because it concerns water whose density is equal to 1 g.cm^−3^. The LAC sorption capacities were subsequently calculated. The result obtained for water stems from an encumbrance of the hydrophobic sorbent during a period of 24 h. For the purposes of comparison, a restatement of the volume of the absorbed liquid per mass of the observed sample was made (Table 6).

A comparison of the experimental data with the data given by the manufacturer for the substrate oil (Table 4) shows a dissimilarity. Experimental values are stated for all cases below.

**Table 4:** Experimental results and a calculation of AC for oil according to EN ISO 9073.

Textile sorption mats	Area of sample (cm^2^)	Average mass of dry sample (g)	Amount of absorbed oil (m_n_-m_k_) (g)	(m_n_-m_k_) in ml	(m_n_-m_k_) given by the manufacturer (Technical sheets)	LAC	LAC given by the manufacturer (Technical sheets)
	Sorbate: oil, oil density = 0,95 g.cm^−3^						
HSR	100	2.71 ± 0.14	21.84 ± 0.97	22.98	18 mL/1 g	10.75 ± 0.25	18
ChSR		1.84 ± 0.08	13.21 ± 0.44	13.9	19.3 mL/1 g	9.55 ± 0.44	19.3
USR		2.95 ± 0.19	19.79 ± 0.50	20.83	18 mL/1 g	11.74 ± 0.23	18

The usage of sorption materials is applied for the purposes of eliminating the effects of dangerous substances and cleaning the environment. The sorbent itself is considered to be ecologically harmless (47–49, 55–57). The Ministry of Environment of the Slovak Republic bestows upon the given types of products the label of “environmentally suitable product” on the basis of fulfilling the required criteria according to the act 469/2002. Textile sorption materials are in the given case divided into universal sorption materials and sorbents with a hydrophobic surface. The merging of ChSR and USR is valid, LAC values are relatively comparable (Tables 4, 6). In this case, the universal sorption materials are able to absorb liquid substances, including water, from solid surfaces. They can appear in the form of lateral formations (mats, coils, hoses), in the form of flakes or granulate (54).

The statement about individual conditions for the bestowal of the national environmental label for the sorption materials product group (54) specifies the requirements about their minimum sorption ability, which extend to water and oil substances. The representative of oil substances is the car gasoline SUPER 95 (Table 5).

**Table 5:** Experimental results and a calculation of AC for gasoline according to EN ISO 9073 compared to Ministry of the Environment of the Slovak Republic (54).

Textile sorption mats	Area of sample (cm^2^)	Average mass of dry sample (g)	Amount of absorbed gasoline (m_n_-m_k_) (g)	LAC	LAC (Ministry of the Environment of the Slovak Republic) (48)
HSR	100	2.82 ± 0.13	21.75 ± 1.03	7.72 ± 0.41	5
ChSR		1.82 ± 0.03	13.26 ± 0.44	7.14 ± 0.25	6
USR		2.9 ± 0.09	19.85 ± 0.53	6.84 ± 0.12	

Universal sorption materials must reach a minimum sorption ability of 6 g of sorbate/1 g of sorbent established according to the EN ISO 9073-6:2004 technical norm while using the medium – car gasoline SUPER 95. The given requirement is met (Table 5). The given requirement is met also for HSR (Table 5). Other specific requirements for minimum sorption ability are also met (Table 7).

**Table 7:** Experimental results of LAC for mats, obtained according to STN EN ISO 9073-6 compared to Ministry of the Environment of the Slovak Republic (54).

Sorbate	LAC					
	HSR		ChSR		USR	
	Experimentally established	Ministry of the Environment of the Slovak Republic (48)	Experimentally established	Ministry of the Environment of the Slovak Republic (48)	Experimentally established	Ministry of the Environment of the Slovak Republic (48)
Water	0.17 ± 0.03	0.5	9.34 ± 0.95	X	9.93 ± 0.18	X
Alcohol	7.79 ± 0.17	X	7.18 ± 0.25	X	8.64 ± 0.19	X
Oil	10.75 ± 0.25	7	9.55 ± 0.44	8	11.74 ± 0.23	8
Gasoline	7.72 ± 0.41	5	7.14 ± 0.25	6	6.84 ± 0.12	6

The results presented thus far are results of sorption under 1 min in duration. The time of saturation of products defined for all sorption materials for oil and chemical substances according to Ministry of the Environment of the Slovak Republic (54) cannot exceed 3 min. Whether the given reality was met was able to be monitored through another method (46).

### 3.2. Evaluating textile sorbents according to ASTM F 726

**Figure 5:**
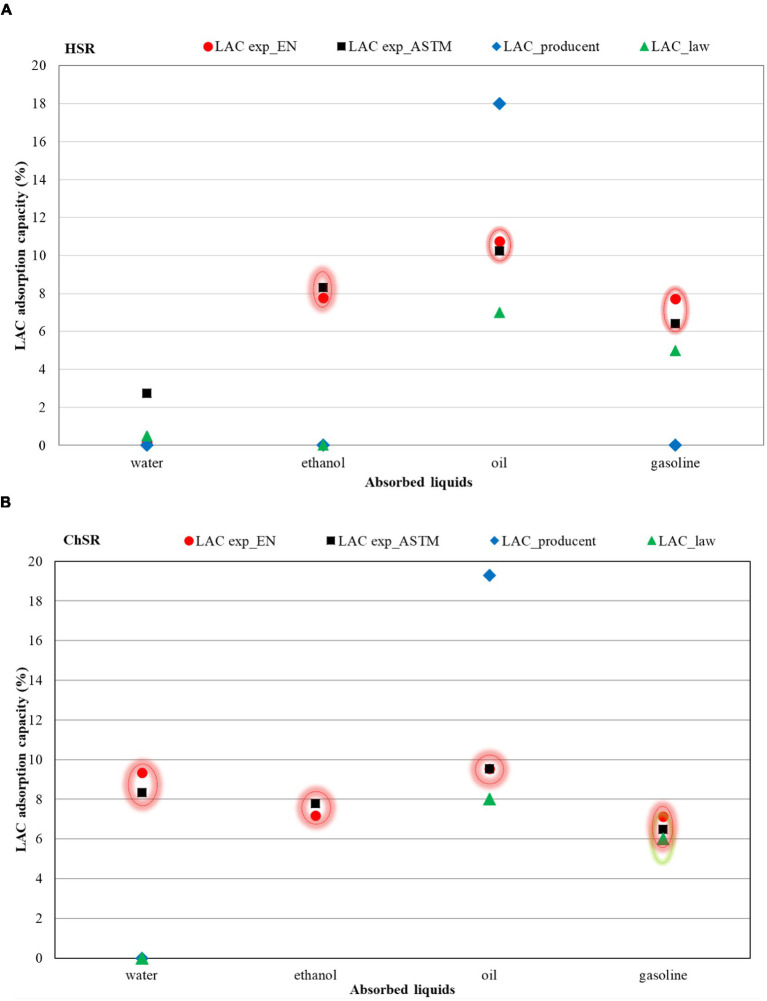

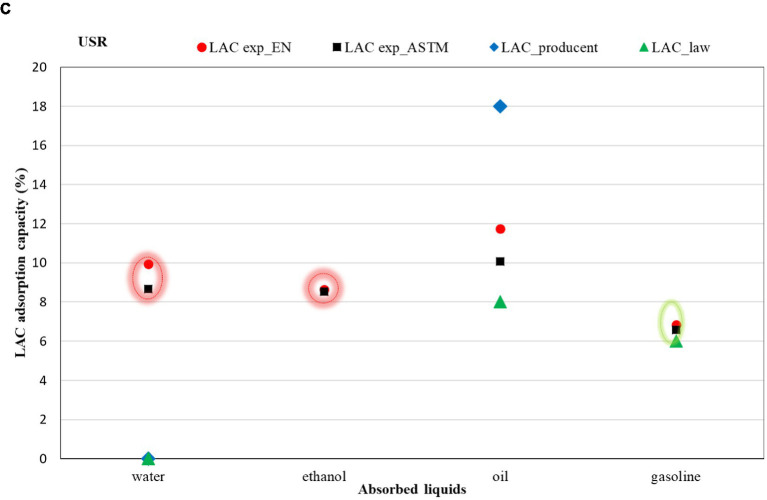
A comparison of experimental data obtained from both methods with the data provided by the manufacturer and an established Slovak Legislation Hydrophobic. **(A)** Sorption mat. **(B)** Chemical sorption mat. **(C)** Universal sorption mat.

The establishment of sorption capacity of textile sorbents was realized over a period of 24 h (Table 8). A summary of the results of both approaches points to comparable results (Figure 5 and Tables 7, 8).

**Table 8:** Presentation of a mass multiple for time of sorption of 24 h of textile sorption material according to ASTM F 726.

Mats	Adsorption capacity per unit mass			
	Water	Oil	Alcohol	Gasoline
Hydro	2.74 ± 0.24	10.24 ± 0.56	8.3 ± 0.08	6.41 ± 0.50
Chem	8.33 ± 0.29	9.54 ± 0.16	7.78 ± 0.25	6.48 ± 0.13
Uni	8.66 ± 0.15	10.06 ± 0.20	8.54 ± 0.18	6.57 ± 0.18

The given graph implies that time of sorption does not play a role or plays a very insignificant one. Because the results of adsorption capacity during a time of sorption of 24 h and 60 s are significantly comparable, in chemical mats equal. The result is good, because it is necessary to perform an immediate, maximally effective disposal of escaping chemicals. A variability of results shows itself in oil sorption in all sorption mats.

The usability of polypropylene fibers is observed also from the viewpoint of usage in composite materials for the purposes of emergency workers safety (58–60).

Crisis management focuses on readiness, prevention, reaction, and a return to initial state after the crisis. For a proper reaction it is necessary to be ready for negative events. Readiness mitigates the negative impact on the environment, lessens the negative impact of the event and makes the return to the pre-crisis state easier. Security has to be given necessary attention with regard to modern technologies, which should raise its standards (45, 61, 62). Despite that it is impossible to completely eliminate or prevent accidents in companies. In companies manipulating dangerous substances, an escape of the substances can occur due to damage or an accident. A significant deal of attention is therefore necessary to be given to the reaction to the escape of dangerous substances during accidents. Some authors point to the negative impact of dangerous substance leakage on the environment and to the need for a reaction to possible accidents (19, 63).

As a follow-up to the reaction, it is necessary to test various products and to compare real findings with technical sheets and norms. The problematic of appropriate sorbent implementation is a part of many investigations, which aim to determine the right sorbents or substances for the elimination of the negative impact of escaping substances on the environment (64–66). The results and comparison of testing will allow companies to choose a sorbent and to also continue with the reaction after the sorbent can no longer absorb the substances. This is what will allow for a more effective reaction and for lesser negative impact on the environment and the lives of people in the vicinity. In the event of a release to the environment or to watercourses, more effort and special sorption procedures are subsequently required to remove or reduce the negative impact of the released substances in water and the environment (67, 68).

## 4. Conclusion

In today’s constantly evolving age connected with a large amount of technological devices we are at all times exposed to a rising risk of extreme events arising. One of the possible extreme events is the escape of a dangerous substance from the site of a company that manipulates it. To mitigate the negative impact of such an event, different sorbents are used in industrial companies. The article was focused on textile sorbents, which can, owing to their sorption ability, allow the elimination or mitigation of a negative impact of a possible accident in the company connected with an escape of a liquid dangerous substance. These sorbents were tested and compared with the established values. Based on the obtained results it is possible to state that sorption capacities of the chemical and universal mat for the substrate water are equal and consistent with the data given by the manufacturer. Textile sorption mats also have a comparable sorption capacity. The sorption capacity on the substrate gasoline is the same in all textile sorbents. All compared sorption mats meet the criteria of the “environmentally suitable product” label. An important finding is the maximum sorption output of the mats, which reached up to 60 s, after which the sorption capacity values remain unchanged, which we consider as a novelty in our research. This fact is very important in the case of emergency services intervention, because this fact is not mentioned by the manufacturers. The responding forces need to know that the said material only performs its task for 60 s. If it does not absorb the entire volume of the spilled liquid within 60 s, it has to be replaced by a new sorbent. Based on the findings it is necessary to adopt further measures to eliminate negative impact in the case of an escape of a dangerous substance that lasts longer than the aforementioned period of time.

The application of the above materials remains relevant. The production, transport and use of hazardous substances remains a part of life and the risk of release of these materials is permanently present. It is necessary to search for suitable sorption materials for the capture of leaked chemicals with the highest possible sorption capacity value, possibly in the manner presented by us.

We are looking for means to quickly and efficiently capture leaked hazardous substances through internationally recognized testing methods. Further research is needed in this topic, as well as above mentioned testing methods in terms of the amount of sample tested and the specific procedure in order to obtain data that would be usable by emergency services (e.g., firefighters) in dealing with the situation.

## Data availability statement

The original contributions presented in the study are included in the article/supplementary material, further inquiries can be directed to the corresponding author.

## Author contributions

IM: Conceptualization, Data curation, Investigation, Methodology, Project administration, Resources, Software, Supervision, Validation, Visualization, Writing – original draft, Writing – review & editing. JK: Conceptualization, Formal analysis, Funding acquisition, Investigation, Methodology, Project administration, Resources, Validation, Visualization, Writing – original draft, Writing – review & editing. ZŠ: Conceptualization, Data curation, Formal analysis, Investigation, Resources, Validation, Writing – original draft. KP: Conceptualization, Formal analysis, Investigation, Methodology, Resources, Validation, Visualization, Writing – original draft, Writing – review & editing.
